# Macrolactonization of methyl 15-hydroxypentadecanoate to cyclopentadecanolide using KF-La/γ-Al_2_O_3_ catalyst

**DOI:** 10.1098/rsos.211479

**Published:** 2022-09-07

**Authors:** Haijun Cheng, Chang Yu, Hongyun Wang, Xiongmin Liu, Li Ma, Fang Lai

**Affiliations:** School of Chemistry and Chemical Engineering, Guangxi University, Nanning Guangxi 530004, People's Republic of China

**Keywords:** KF-La/γ-Al_2_O_3_, high basic strength, methyl 15-hydroxypentadecanoate, macrolactonization, cyclopentadecanolide

## Abstract

It has been a challenge to synthesize macrolide musk in excellent yields with high purity. KF-La/γ-Al_2_O_3_ catalyst was prepared from a highly basic mesoporous framework using a mild method. The prepared KF-La/γ-Al_2_O_3_ catalyst was employed for the synthesis of cyclopentadecanolide from methyl 15-hydroxypentadecanoate. The morphology and structure of prepared catalysts were characterized using XRD, TG-DTG, SEM, EDX, TEM, BET and CO_2_-TPD. The results revealed that the K_3_AlF_6_ and LaOF are produced on the surface of KF-La/γ-Al_2_O_3_, and LaO can promote the dispersion of KF on the surface of Al_2_O_3_. Catalysts pore size main distribution ranges between 10 and 30 nm, the maximum CO_2_ desorption temperature is 715°C when the La loading is 25%. Because F^−^ ion has a higher electronegativity than O^2−^ ion, the KF-promoted metal oxide (Al_2_O_3_ or/and La_2_O_3_) contained more strong basic sites, compared with that of the corresponding metal oxide. The yield of cyclopentadecanolide obtained at 0.5 g KF-25La/γ-Al_2_O_3_ catalyst and a reaction temperature of 190°C for 7 h were 58.50%, and the content after reactive distillation is 98.8%. The KF-La/γ-Al_2_O_3_ catalyst has a larger pore size and basic strength, which is more conducive to the macrolactonization of long-chain hydroxy ester.

## 1. Introduction

Macrolactones are lactones containing more than eight atoms in the lactone ring. The structural motifs of macrolactone are widely observed in natural products, drug molecules and bioactive molecules [1,2]. Macrolactones are commonly used to produce biological pheromones, drugs [3–6], pesticides [7], antimicrobial agents and spices [8]. Macrolactones containing 15–17 carbon atoms have attracted significant attention in the cosmetic industry as a substitute for natural musks because of their unique fragrance. This could be attributed to the fact that they do not display the cancerogenic and bioaccumulation toxic properties [9–11] associated with nitro-musks and polycyclic-musks. In addition, the cost of synthesizing macrolactones is significantly lower than that of extracting natural musk. Consequently, in the past decades, macrolactonization reactions have attracted increased attention among researchers.

Macrolides can be artificially synthesized using chemical methods [12]. It is well known that long-chain hydroxy fatty acids do not readily form macrolides compounds via macrolactonization owing to the binding of reactive molecules, which leads to the formation of dimers or oligomers. Therefore, these methods require the use of catalyst to increase the macrolides yields [13]. The chemical synthesis of macrolactone is a cost-effective procedure [14–16]. Conventional methods for macrolide synthesis typically include: a direct macrolactonization of the corresponding seco-acid [14,15,17–20], ring-closing metathesis [21,22], cross-coupling reactions [23–26], macrolactonization of alkenyl acid, macrocyclic olenation reactions [27] and catalytic carbonylative macrolactonization [28,29]. Additionally, catalytic C–H macrolactonization [18,30], enantioselective Rh-catalysed redox-neutral allene-acid macrocyclization [31] and NHC-catalysed oxidative macrolactonization [32] as an unconventional method have also been explored. Currently, direct macrolactonization of the corresponding seco-acid still remains the most prevalent method. In 1985, Boden & Keck used ω-hydroxy acids of various chain lengths as the raw material 4-dimethylaminopyridine (DMAP) as the catalyst to react in dicyclohexylcarbodiimide (DCC) solution for 16 h to synthesize the corresponding macrolide compound, and the product was isolated and purified, which affords a 95% isolated yield of hexadecanolide [16]. Mukaiyama *et al*. prepared medium-sized lactones on treating monomeric cyclic silyl siloxycarboxylates, prepared *in situ* from ω-hydroxycarboxylic acids and 1,2-bisdimethylsilylbenzeneusing RhCl(PPh_3_)_3_ catalyst, with an active catalyst of dimethylsilylbis (trifluoromethanesulfonate) [33]. In this experiment, 10 ml of benzene was added as a solvent, and the amount of ω-hydroxy acids added was 0.2 mmol. After 18 h of reaction, column chromatography on silica to afford lactone, the highest yield was 87%. Shiina *et al*. [34] described an efficient method for the synthesis of various esters, involving the intermediary formation of a mixed anhydride with benzoic acid anhydrides in the presence of catalytic amounts of Lewis acids. TiCl_4_, toluene and chlorotrimethylsilane were used as active reagents, and dichloromethane was used as solvent. Finally, the crude product was purified by thin layer chromatography and the highest yield of macrolide was 83%. Ookoshi & Onanka reported the macrolactonization of ω-hydroxyalkanoic acid is catalysed by dealuminated HY zeolite in a concentrated toluene solution [35]. The highest yield of macrolide was 51% after 24 h of reaction. De Léséleuc & Collins used Hf(OTf)_4_ to catalyse the direct macrolactonization of seco-acid (5 mmol l^−1^) in the presence of toluene as a solvent, and the highest macrolide yield of 87% was obtained at 100°C for 24 h [36]. However, there are several inherent limitations associated with traditional seco-acid methods. For example, a stoichiometric amount of activating reagents are required for the activation of either the acid or the hydroxyl group [3]. The reaction time is excessively long, whereas the intermolecular synthetic processes always occur along with competitive reactions, leading to the undesired polymer as by-products [37]. In addition, to minimize intermolecular dimerization, highly diluted conditions or slow addition protocols are required [38,39] and selectivity in the closed-loop reaction step is poor [40], which has the low synthetic efficiency and economy. The reagents used in the experiment are highly toxic, especially the liquid catalysts which are prone to corrode the equipment, make post-treatment difficult and pose a threat to the environment.

Although Ookoshi & Onanka [35] and Lai *et al.* [37] used solid acid as catalyst, a large amount of organic solvent was added during the reaction, and the product needed to be purified. To the best of our knowledge, the development of heterogeneous solid base catalysts has attracted significant attention in recent years [41,42]. For example, solid base catalysts have been widely employed in transesterification owing to its eco-friendliness and remarkable activity [43–47]. We can roughly divide solid base catalysts into two types. One is an unsupported solid base, which includes alkaline earth metal oxides, rare earth oxides and composite oxides. The other is supported solid bases, namely alkali metal oxides and hydroxides supported on various porous materials, etc. [48].

The catalytic activity of a solid base catalyst is determined by its solid structure and surface characteristics, particularly, its basicity and specific surface area. Usually, in order to enhance base strength, alkaline-earth oxide, rare-earth oxide and their mixed oxides frequently modified with potassium compounds, including KF, K_2_CO_3_, KOH and K_2_O [49–52]. KF-loaded metal oxides exhibit high catalytic activity to macrolactonization [53]. La_2_O_3_ is widely used as a basic carrier or catalytic promoter owing to its low polarizing power [54]. Although KF-supported La_2_O_3_ exhibits strong basicity, it has the disadvantages of high calcination temperature, small specific surface area and low selectivity. To obtain a solid base catalyst with a large specific surface area [55,56], Liu *et al*. [57] used La_2_O_3_ as a protection medium for SBA-15 support. The combination of KF and La_2_O_3_ facilitated the generation of super basic sites to obtain a solid base catalyst with an average pore diameter of approximately 5 nm. However, the high cost and relatively small average pore size of SBA-15 support limit its application in catalysing macromolecular species reactions [35]. Therefore, the development of supported solid superbases is beneficial to catalyse macrolactonization reactions.

Among various supports, γ-Al_2_O_3_ has attracted significant attention among researchers as a mesoporous material [58,59] owing to its large specific surface area, favourable dispersibility, thermostability [60] and high catalytic performance [61,62]. The addition of La is known to stabilize alumina for high temperature applications and Lanthanum acts as a promoter in combination with Al_2_O_3_ to render the solid strongly basic [63,64]. Considering that methyl 15-hydroxypentadecanoate is a long-chain macromolecule containing 16 carbon atoms. In this study, a modified wetness impregnation method was used to sequentially load La_2_O_3_ and KF on the surface of γ-Al_2_O_3_ at mild calcination temperature, which avoided the decomposition of nitrate anion at high temperature brought by potassium. The synthesized strong basic KF-La/γ-Al_2_O_3_ solid catalyst exhibited a large specific surface area and pore size [65,66]. The structure, surface area, basicity and thermal stability of the prepared KF-La/γ-Al_2_O_3_ mesoporous solid base catalyst were characterized using XRD, TG-DTG, SEM, EDX, TEM, BET and CO_2_-TPD. Methyl 15-hydroxypentadecanoate ester was used as the raw material for the macrolactonization. Under the presence of the synthesized KF-La/γ-Al_2_O_3_ solid base, a high value-added musk containing cyclopentadecanolide was obtained. The formation of the macrolide does not require more than stoichiometric amounts of reagents to activate the carboxylate or the alcohol. In this study, we discussed the formation process of the strong basic sites in the KF-La/γ-Al_2_O_3_ catalyst, and researched the influence of the structure and morphology of the catalyst and the macrolactonization of long-chain hydroxy ester and optimized the reaction conditions.

## 2. Material and methods

### 2.1. Chemicals

*Malania Oleifera Chum* oil was obtained from Yandong Township Bama County Guangxi province in China. Diethyl ether and sulfuric acid were purchased from Chengdu Kelong Chemical Co., Ltd (Chengdu, China). Methanol and ethanol were purchased from Xilong Scientific Co., Ltd (Guangdong, China). n-hexane and glycerine were purchased from Guangdong Guanghua Sci-Tech Co., Ltd (Guangdong, China). Cyclopentadecanolide (greater than 98%) was purchased from Sigma-Aldrich Co., γ-Al_2_O_3_ (purity greater than 99.9%), lanthanum nitrate hexahydrate and 2-amino-2-methyl-1-propanol were purchased from Shanghai Aladdin Biochemical Technology Co., Ltd (Shanghai, China). Potassium fluoride was purchased from Shanghai Macklin Biochemical Co., Ltd (Shanghai, China). All reagents are analytical grade.

### 2.2. Catalyst preparation

KF-La/γ-Al_2_O_3_ was synthesized according to the reported method [67] as follows: La_2_O_3_ was introduced to γ-Al_2_O_3_ using the incipient wetness impregnation method. Typically, γ-Al_2_O_3_ was roasted in a muffle furnace at 550°C for 3 h. Simultaneously, 0.78 g of La(NO_3_)_3_ · 6H_2_O was dissolved in 40 ml ethanol, after which 1.0 g of γ-Al_2_O_3_ was added into the mixture. Subsequently, the resulting mixture was stirred at 40°C for 24 h, after which the mixture was heated and stirred in an oil bath at 60°C until the solvent evaporated. Thereafter, the obtained solid was dried at 60°C overnight. Lastly, the precursor was calcined in a muffle furnace at 550°C for 4 h to convert La(NO_3_)_3_ to La_2_O_3_. The resulting γ-Al_2_O_3_-supported La oxides were denoted as (*ω*)La/γ-Al_2_O_3_, where *ω* is the La/Al_2_O_3_ mass ratio.

KF was introduced to the surface of the as-synthesized (*ω*)La/γ-Al_2_O_3_ using the wetness impregnation method. First, 0.2 g of KF was dissolved in 40 ml of absolute methanol, after which (*ω*)La/γ-Al_2_O_3_ was added to the mixture. Subsequently, the mixture was stirred at room temperature until the solvent evaporated, after which the precipitate was dried for 24 h under vacuum at 60°C. The obtained catalyst precursor was calcined in a muffle furnace at 350°C for 4 h, during which KF was activated, leading to the formation of KF-(*ω*)La/γ-Al_2_O_3_. KF/γ-Al_2_O_3_ and La/γ-Al_2_O_3_ were also synthesized using the aforementioned methods.

### 2.3. Synthesis of methyl 15-hydroxypentadecanoate

Methyl 15-hydroxypentadecanoate was synthesized using the following method. First, *Malania Oleifera Chum* oil was subjected to saponification, acidification, and solvent crystallization to obtain 15-tetracosenoic acid. Subsequently, 30.0 g of 15-tetracosenoic acid was added to a solution composed of 40 ml of ethanol and 160 ml of n-hexane. The mixed solution was placed in a thermostatic water bath at 0°C, after which the mixture was exposed to ozone generated using a laboratory-scale corona discharge generator continuously bubbling into the reactor. A change in the colour of a wet starch potassium iodide test paper to blue was used to indicate the end of the ozonation reaction. The obtained ozonide intermediate was added into a beaker, after which potassium borohydride solution was slowly added into the beaker and continuously stirred for 3 h. Lastly, 6 mol l^−1^ of hydrochloric acid was added to the solution until the pH value of the solution was 2, after which the solution was filtrated, washed to neutrality with deionized water and dried under vacuum at 70°C to obtain 15-hydroxypentadecanoic acid.

The prepared 15-hydroxypentadecanoic acid was purified using 2-amino-2-methyl-1-propanol to obtain an amine compound. Subsequently, the compound was subjected to methyl esterification reaction by dissolving 15 g of 15-hydroxypentadecanoic acid in 300 ml of methanol, after which 2 ml of concentrated sulfuric acid was added into the mixture. The obtained mixed solution was reacted in a thermostatic oil bath at 90°C for 4 h in a reflux device. After the reaction, the mixed solution was extracted using ether, and then washed with deionized water. The solvent in the upper layer of the solution separated from the mixed solution was removed using a rotary evaporator under vacuum condition at 70°C. Lastly, methyl 15-hydroxypentadecanoate with an average content of 90% was obtained.

### 2.4. Catalysts characterization

The XRD patterns of the samples were recorded using a Rigaku Ultima IV diffractometer with monochromatic Cu K*_α_* radiation (*λ* = 1.5418 nm) in the 2*θ* ranges of 10–80°.

TGA analysis was performed using a TA Instruments Q500 Thermogravimetric Analyzer. To prepare the TGA sample, 10.0 mg of the catalyst was placed in a sample pan, after which the catalyst was heated from 30 to 750°C at a rate of 10°C min^−1^ under nitrogen atmosphere. The TGA analysis was performed under nitrogen atmosphere, and the total flow rate was maintained at 35.0 ml min^−1^.

The microscopic morphology and surface structure of the catalysts were characterized using TEM (FEI Talos F200 s, USA), and the chemical composition of the catalysts was examined using SEM-EDX (Hitachi SU-5000, Japan).

The specific surface area, pore-volume and pore size of the catalysts were measured using a NOVA 2200e gas sorption analyser (Quantachrome Company, Boynton Beach, FL, USA) via N_2_-physisorption adsorption and desorption at −196°C. Before testing, the catalyst was degassed under nitrogen atmosphere at 120°C for 2 h. The specific surface areas of the catalysts were calculated using the Brunauer–Emmett–Teller (BET) method, and the pore volume and diameter were calculated using the Barrett–Joyner–Halenda (BJH) model.

CO_2_-TPD was used to measure the total basicity and basic strength distribution of the catalyst using a FINESORB-3010 apparatus equipped with a thermal conductivity detector. To prepare the sample used for the analysis, 50.0 mg of the catalyst samples (40–60 mesh) were pretreated at 110°C under helium flow (99.999%) at a rate of 10 ml min^−1^ for 1 h, and the samples were saturated with pure CO_2_ flow after cooling to room temperature. Subsequently, the pretreated samples were purged in helium atmosphere at room temperature until the baseline was sufficiently stable to remove physisorbed CO_2_. Thereafter, the samples were heated from room temperature to 850°C at 10°C min^−1^ under helium flow.

### 2.5. Evaluation of catalytic performance

The as-synthesized methyl 15-hydroxypentadecanoate was used for the synthesis of cyclopentadecanolide. Typically, methyl 15-hydroxypentadecanoate (5 mmol), KF-La/γ-Al_2_O_3_ catalyst (0.5 g) and glycerine (15 ml) were mixed and stirred for 30 min at 120°C using an electric heater with magnetic stirring. Subsequently, the system was slowly heated to 190°C under 2 mbar pressure and maintained for 7 h. The produced cyclopentadecanolide was separated from the reaction system using the reaction–distillation method through an oil–water separator. The reaction–distillation reaction shifted the chemical equilibrium in favour of the formation of cyclopentadecanolide. The cyclopentadecanolide crystals floating on the surface of glycerol were collected.

### 2.6. Analysis of products

Methyl 15-hydroxypentadecanoate and cyclopentadecanolide were analysed using Ultra gas chromatography mass spectrometry (GC-MS, Shimadzu GCMS-QP2010). To calculate the yield of the target products, the products were detected using gas chromatography (Shimadzu GC-2010 Plus). The cyclopentadecanolide product yield was calculated as follows:

$$\mathrm{yield}\;(\text{\%})=\frac{m(\mathrm{product})}{m(\mathrm{theory})}\times 100\text{\%}$$

## 3. Results and discussion

### 3.1. Characterization of the catalyst

#### 3.1.1. Structure of the catalyst

**Figure 1.**
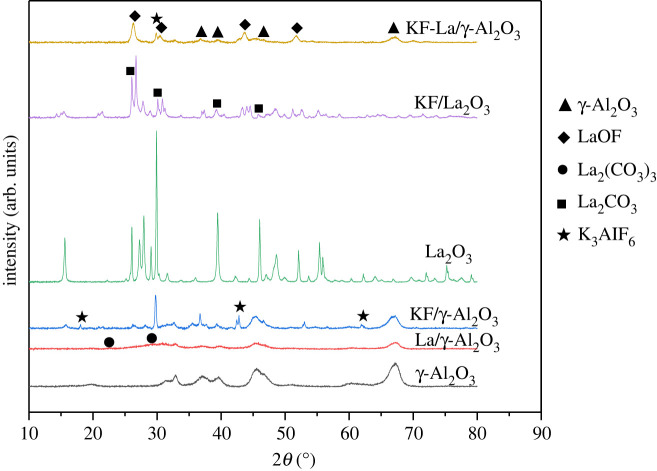
X-ray diffraction patterns of the different synthesized catalysts.

The XRD patterns of the γ-Al_2_O_3_, La/γ-Al_2_O_3_, KF/γ-Al_2_O_3_, KF-La/γ-Al_2_O_3_ and KF/La_2_O_3_ catalysts are shown in figure 1. Distinct characteristic peaks were observed in the XRD patterns of the pure γ-Al_2_O_3_ with significant intensity at 2*θ* = 36.8, 39.6, 46.5 and 67.2° (PDF# 49-0134).

However, no clear and sharp diffraction peaks associated with the supported metal crystallites were observed in the XRD pattern of the La/γ-Al_2_O_3_ samples. In addition, the intensity of the diffraction peak of γ-Al_2_O_3_ was significantly weakened. This suggests that the introduced La species were evenly dispersed on the γ-Al_2_O_3_ support as amorphous phases or nanocrystals. Characteristic peak of La_2_(CO_3_)_3_ (PDF# 25-1400) were observed in the XRD pattern of La/γ-Al_2_O_3_ at 2*θ* = 22.5 and 29.5°, which could be attributed to the fact that La_2_O_3_ readily reacts with CO_2_ in air to form La_2_(CO_3_)_3_. Diffraction peaks were observed in the XRD pattern of the KF/γ-Al_2_O_3_ catalyst at 2*θ* = 18.0, 29.7, 42.8 and 62.0° (PDF# 03-0615), which could be attributed to the presence of K_3_AlF_6_ in the sample. In addition, the intensity of the γ-Al_2_O_3_ peak decreased, which could be attributed to the fact that the surface of γ-Al_2_O_3_ was covered with KF and K_3_AlF_6_. In addition to the diffraction peaks of K_3_AlF_6_ and Al_2_O_3_, new diffraction peaks were observed in the XRD pattern of KF-(*ω*)La/γ-Al_2_O_3_ at 2*θ* = 26.7, 30.5, 44.1 and 51.8°(PDF# 77-0204), which could be attributed to the presence of LaOF. Furthermore, only a small peak corresponding to K_3_AlF_6_ was observed in the XRD pattern of the KF-La/γ-Al_2_O_3_ catalyst at 2*θ* = 29.7°, and other characteristic peaks disappeared. This could be attributed to the fact that: (i) the uniform covering of La_2_O_3_ on the surface of γ-Al_2_O_3_ hindered the reaction between KF and γ-Al_2_O_3_; (ii) most of the KF reacted with La_2_O_3_ to form LaOF, thus diffraction peak of La_2_(CO_3_)_3_ or La_2_O_2_CO_3_ were not observed in the XRD pattern of the KF-La/γ-Al_2_O_3_ catalyst. This indicates that the introduction of KF improved the CO_2_ resistance of the La/γ-Al_2_O_3_ catalyst and prevented the consumption of the basic site by acid gases, such as CO_2_ [68]. In addition, peaks relating to KF were not observed, which could be attributed to the high dispersion of KF on the surface of the catalyst. These findings indicate that the γ-Al_2_O_3_ of the KF-(*ω*)La/γ-Al_2_O_3_ catalyst was sequentially covered with La_2_O_3_ and KF, and a new compound (i.e. LaOF) was formed on the outermost layer.

**Figure 2.**
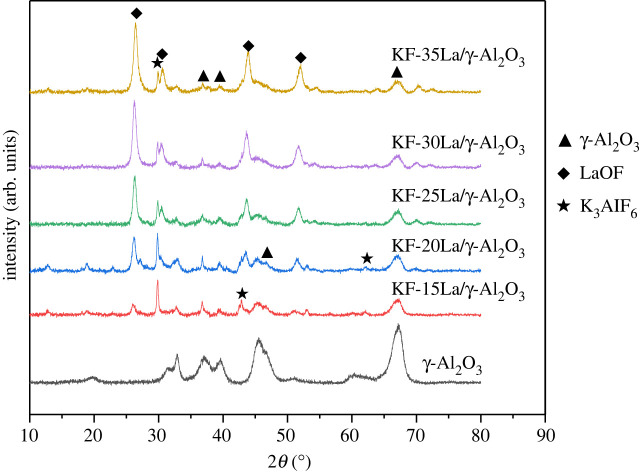
X-ray diffraction patterns of the KF-La/γ-Al_2_O_3_ catalysts with different La loadings.

The XRD patterns of the KF-(*ω*)La/γ-Al_2_O_3_ (*ω* = 15, 20, 25, 30 and 35%, where *ω* is the La/Al_2_O_3_ mass ratio) catalysts with different La loading are shown in figure 2. The major diffraction peaks in the XRD patterns of the samples are the peaks of LaOF, K_3_AlF_6_ and Al_2_O_3_. In addition, the XRD results revealed that the intensity of the characteristic peaks of LaOF gradually increased with an increase in the La species loading in γ-Al_2_O_3_, whereas the strength of the characteristic peaks of K_3_AlF_6_ (2*θ* = 29.7, 42.8, 62.0°) reduced. This indicates that an increase in the La loading content facilitated the formation of LaOF, as the La species hindered the reaction between KF and Al_2_O_3_. It can be concluded that both La_2_O_3_ and KF have been supported on γ-Al_2_O_3_, and LaOF metal salts are formed on the surface, which may have a direct impact on the catalytic macrolactonization.

#### 3.1.2. TG-DTA results of the La/γ-Al_2_O_3_ before and after calcination

**Figure 3.**
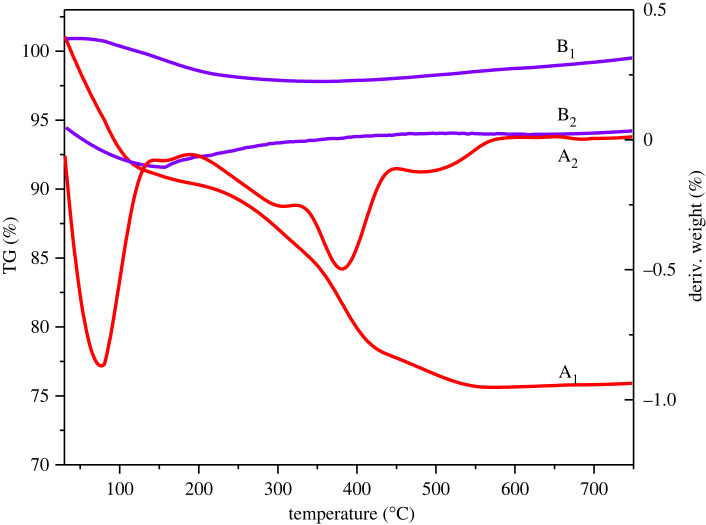
TG and DTG curves of the La/γ-Al_2_O_3_ before (A1 and A2) and after (B1 and B2) calcination.

The TG and DTG curves of the as-prepared La/γ-Al_2_O_3_ before and after calcination are shown in figure 3. The first weight loss of the uncalcined La/γ-Al_2_O_3_ was observed between 30°C and 150°C, which could be attributed to the removal of the methanol solvent and water molecules. The second weight loss was observed between 330°C and 550°C, which could be attributed to the conversion of lanthanum nitrate to lanthanum oxide. In contrast, only one weight loss was observed in the DTG curves of the calcined La/γ-Al_2_O_3_ catalyst at approximately 150°C, which could be attributed to the removal of water molecules. In addition, there was no significant weight loss after 230°C, indicating the complete conversion of the lanthanum nitrate on the surface of the La/γ-Al_2_O_3_ catalyst to lanthanum oxide. These results indicate that the optimum calcination temperature was 550°C.

**Figure 4.**
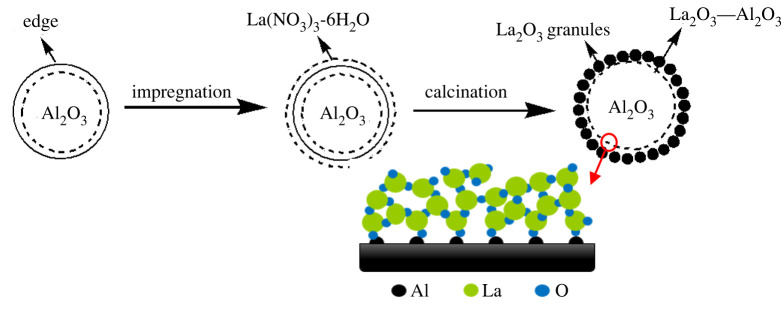
Proposed formation process of La/γ-Al_2_O_3._

During calcination, La-O species attached to Al–O species to form a dense layer on the surface of the γ-Al_2_O_3_ support [69]. Consequently, this enhanced the even dispersion of La species on the surface, as confirmed by the XRD results. The possible dispersion of La_2_O_3_ on Al_2_O_3_ is shown in figure 4.

#### 3.1.3. Morphology and composition of the catalysts

**Figure 5.**
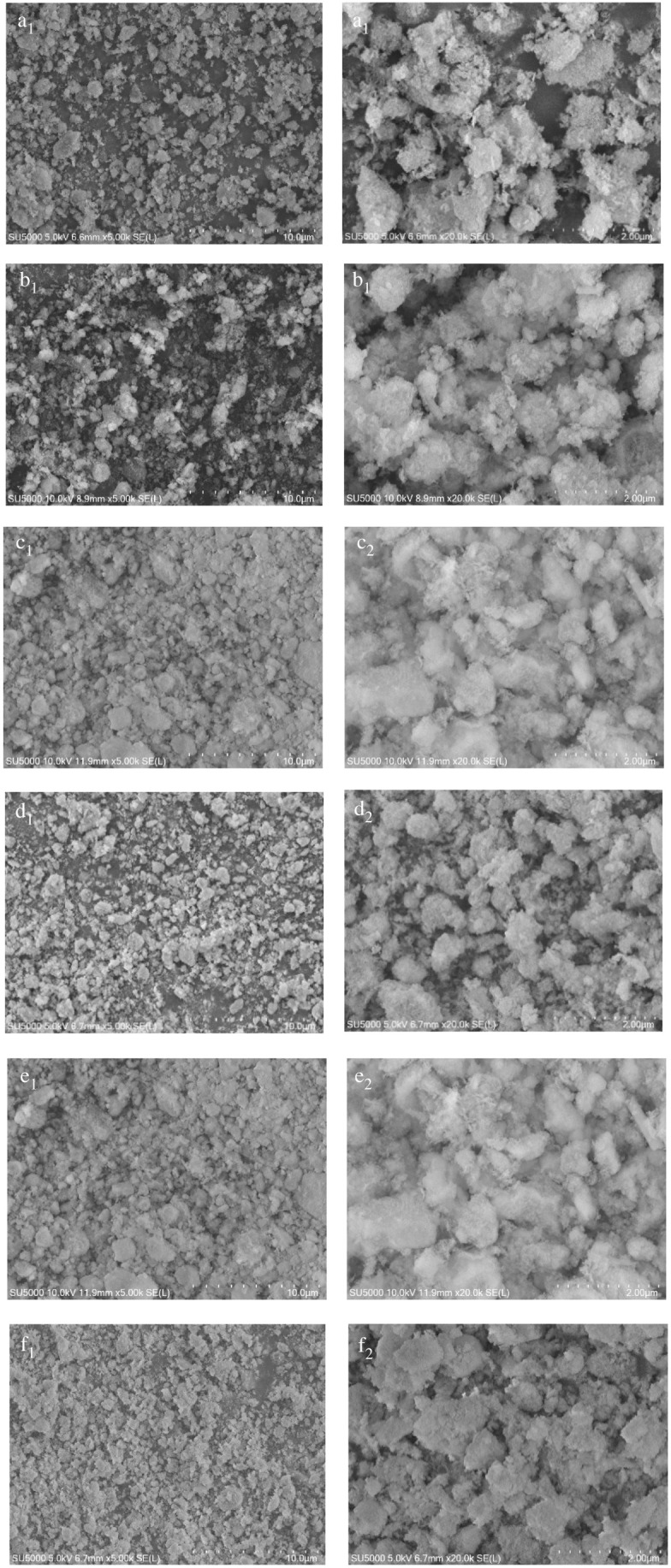
SEM images of (*a*) γ-Al_2_O_3_, (*b*) KF-15La/γ-Al_2_O_3_, (*c*) KF-20La/γ-Al_2_O_3_, (*d*) KF-25La/γ-Al_2_O_3_, (*e*) KF-30La/γ-Al_2_O_3_ and (*f*) KF-35La/γ-Al_2_O_3_.

The morphology of the KF-(*ω*)La/γ-Al_2_O_3_ catalysts with different La loadings was characterized using SEM, and the results are shown in figure 5. The SEM images revealed that γ-Al_2_O_3_ exhibited a non-spherical morphology and irregular particles with a size of 1–10 µm (figure 5*a*_1_). In addition, the particles exhibited a rough surface (figure 5*a*_2_). Furthermore, a comparison of figure 5*b*_1_–*f*_1_ and figure 5*b*_1_,*c*_1_,*e*_1_,*f*_1_ reveals the presence of notable agglomeration on the surface of the particles, which is consistent with the high-magnification SEM image in figure 5*b*_2_,*c*_2_,*e*_2_,*f*_2_. As shown in figure 5*b*,*c*, agglomeration can be observed on the surface of the KF-15La/γ-Al_2_O_3_ and KF-20La/γ-Al_2_O_3_ catalysts, which could be attributed to the reaction between KF Al_2_O_3_. With an increase in the La loading to 35%, several agglomerated particles were observed on the surface of the KF-30La/γ-Al_2_O_3_ and KF-35La/γ-Al_2_O_3_ catalyst (figure 5*e*_1_,*f*_1_), thus giving the surface of the catalyst a smooth appearance (figure 5*e*_2_,*f*_2_). This could be attributed to the excessive accumulation of La_2_O_3_. However, there was no change in the particle size of the KF-25La/γ-Al_2_O_3_ catalyst as there was no agglomeration of particles, and the surface roughness was high (figure 5*d*_1_,*d*_2_). This may be related to the uniform coverage of KF and La_2_O_3_ on the surface of γ-Al_2_O_3_, which is consistent with the XRD and EDX results. At a La loading of 25%, the catalyst exhibited a relatively good morphology.

**Figure 6.**
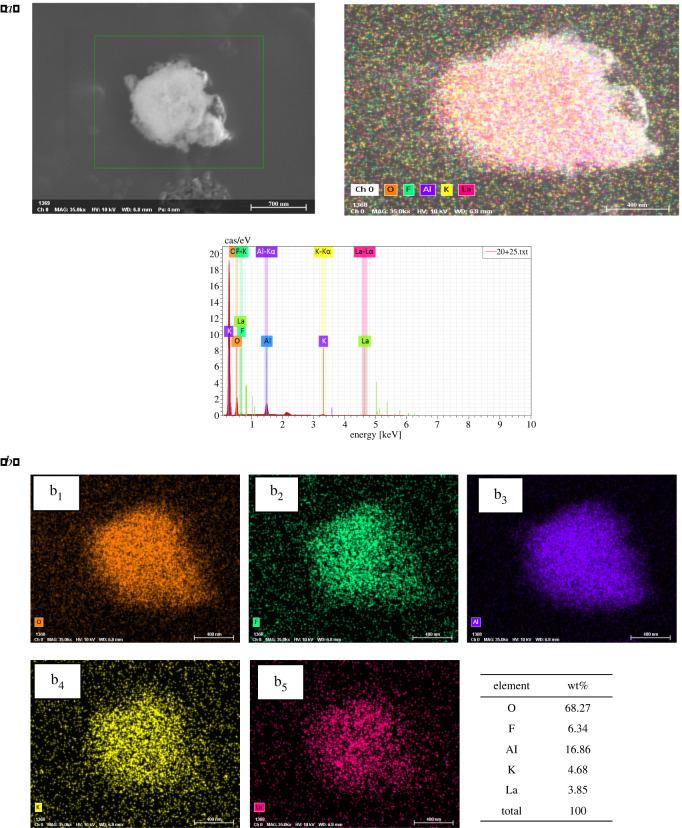
(*a*) SEM image for EDX mapping site and elemental spectrum for KF-La/γ-Al_2_O_3_ catalyst. (*b*). EDX elemental mapping of (*b*_1_) O, (*b*_2_) F, (*b*_3_) Al, (*b*_4_) K and (*b*_5_) La of the KF-La/γ-Al_2_O_3_ catalyst.

The elemental composition and distribution of the KF-La/γ-Al_2_O_3_ catalyst were further investigated using EDX analysis, and the results are shown in figure 6*a*,*b*. For the EDX analysis, an area on the catalyst surface was selected, and the mass percentage of each element was measured. The mass percentages of O, F, Al, K and La were 68.27, 6.34, 16.86, 4.68 and 3.85%, respectively. Different colours were used to represent different elements. Al and O had the highest concentration with a relatively concentrated distribution, which could be attributed to the fact that Al_2_O_3_ was used as the support. By contrast, the contents of other elements were relatively low, and they were uniformly distributed on the surface of the support, which is consistent with the XRD results.

**Figure 7.**
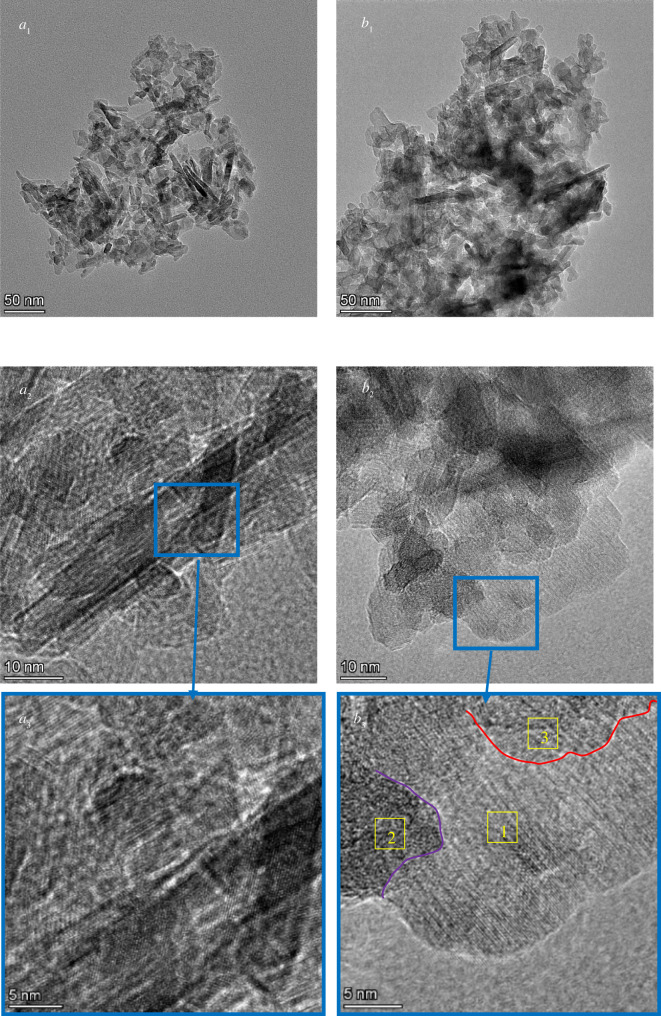
TEM images of the (*a*) γ-Al_2_O_3_ and (*b*) KF-La/γ-Al_2_O_3_ catalysts.

The TEM images of the KF-La/γ-Al_2_O_3_ and γ-Al_2_O_3_ samples at different magnifications are shown in figure 7. The TEM images (figure 7*a*_1_) revealed that there were substantial mesopores inside the γ-Al_2_O_3_ catalyst, which maintained a crack-shaped structure. This is consistent with the BET results. After the γ-Al_2_O_3_ was loaded with KF and La species, its channels became indistinct (figure 7*b*_1_), which could be attributed to the uniform distribution of KF and La_2_O_3_ in the pores and the formation of LaOF on the surface of the catalyst. Figure 7*a*_2_,*a*_3_ shows the characteristic crystal planes and the corresponding lattice fringes of γ-Al_2_O_3_. Different crystal planes and lattice fringes (figure 7*b*_2_) were observed in the TEM image of KF-La/γ-Al_2_O_3_. The lattice fringes can be roughly divided into three regions (figure 7*b*_3_). According to the XRD results, these three regions corresponded to Al_2_O_3_, K_3_AlF_6_ and LaOF. Through the SEM, EDX and TEM analysis of KF-La/γ-Al_2_O_3_, we can clearly see the appearance of KF-La/γ-Al_2_O_3_, as well as the distribution of loaded KF and La_2_O_3_ on γ-Al_2_O_3_. This is advantageous for judging the proper load amount.

#### 3.1.4. Surface area of the catalyst

**Figure 8.**
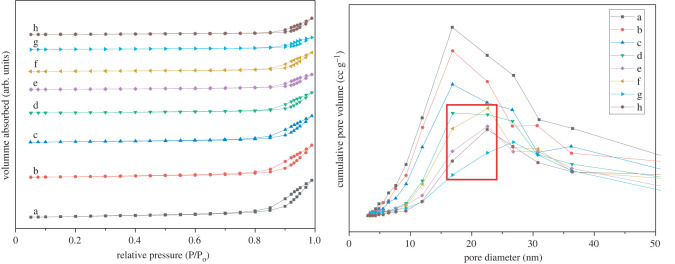
N_2_ adsorption–desorption isotherms and pore size distributions of (a) γ-Al_2_O_3_, (b) KF/γ-Al_2_O_3_, (c) La/γ-Al_2_O_3_, (d) KF-15La/γ-Al_2_O_3_, (e) KF-20La/γ-Al_2_O_3_, (f) KF-25La/γ-Al_2_O_3_, (g) KF-30La/γ-Al_2_O_3_, (h) KF-35La/γ-Al_2_O_3_.

The N_2_ adsorption–desorption isotherms and pore size distribution of the catalysts with γ-Al_2_O_3_ as the carrier are shown in figure 8. Based on the adsorption isotherms classified by the International Union of Pure and Applied Chemistry (IUPAC), the results revealed that all the samples exhibited type IV adsorption–desorption isotherms with an H_3_ hysteresis loop, which is a typical feature of mesoporous materials. This indicates that the catalyst exhibited an irregular slit and crack structure, which is consistent with the structure of the γ-Al_2_O_3_ support, suggesting that the modification had no effect on the original structure of the catalyst.

After loading the γ-Al_2_O_3_ with KF and La_2_O_3_, the initial relative pressure of the hysteresis loops increased from 0.8 to 0.85 (figure 8d–h), and the relative pressure of the capillary condensation in the pores of the catalyst increased. In addition, with an increase in the La loading content, the distance between the adsorption and desorption branches of the catalyst decreased, thus weakening the capillary condensation phenomenon. This indicates that the number of small pores in the catalyst reduced, whereas the numbers of larger-diameter mesopores increased. Because a long-chain hydroxy ester was used as the raw material, the fine pores prevented the entry of the raw material into the pores of the catalyst. However, the small-diameter pores of the catalyst increased diffusion by resisting the entry of raw materials and prolonging the retention time of reactants and products [70]. This facilitated a cracking reaction and carbon deposition during the high-temperature reaction. Consequently, a large number of by-products were produced, thus reducing the selectivity of cyclopentadecanolide. These findings indicate that a reduction in the number of pores has a positive effect on the performance of the catalyst. This suggests that the production of large-diameter pores enhanced the diffusion rate of the hydroxy esters and the migration of molecules, which significantly affected the catalytic performance of the catalyst.

The pore size distribution diagram revealed that all the catalysts exhibited a wide pore size distribution with the main distribution range between 10 and 30 nm. With an increase in the La loading content, the numbers of large-diameter mesopores gradually increased. This could be attributed to the coverage of relatively small mesopores by KF and lanthanum oxide, and the formation of new materials, such as K_3_AlF_6_ and LaOF, which blocked the small mesopores.

Table 1 shows the specific surface area, pore volume and pore diameter of the various catalysts. For the single-load and double-load compounds, the specific surface area and pore volume of the catalyst decreased gradually with an increase in the La loading content. Nevertheless, the average pore diameter of the loaded catalyst was larger than that of pure γ-Al_2_O_3_, due to after the small pores were filled, the large pores dominate. The KF-25La/γ-Al_2_O_3_ catalyst exhibited a relatively large pore volume and the smallest average pore size. Owing to the full coverage of La_2_O_3_, the catalyst exhibited a better protective effect on the medium pore, which could be attributed to the fact that the small pore was filled and the high dispersion of KF on the surface of the catalyst. This was consistent with the XRD results. This shows that the specific surface area and pore size distribution have a huge influence on the catalytic performance of KF-La/γ-Al_2_O_3_.

**Table 1.** Specific surface area, pore volume and pore diameter of the different catalysts.

samples	S_BET_ (m^2^ g^−1^)	V_P_ (cm^3^ g^−1^)	D_P_ (nm)	amount of basic sites (mmol g^−1^)
γ-Al_2_O_3_	134.1	0.69	16.83	0.16
KF/γ-Al_2_O_3_	98.21	0.59	16.88	0.30
La/γ-Al_2_O_3_	86.12	0.49	16.88	0.24
KF-15La/γ-Al_2_O_3_	62.94	0.38	16.92	0.35
KF-20La/γ-Al_2_O_3_	54.86	0.28	22.58	0.41
KF-25La/γ-Al_2_O_3_	50.44	0.35	16.91	0.37
KF-30La/γ-Al_2_O_3_	41.82	0.23	22.56	0.43
KF-35La/γ-Al_2_O_3_	37.32	0.29	22.53	0.46

#### 3.1.5. Basicity of the catalyst

**Figure 9.**
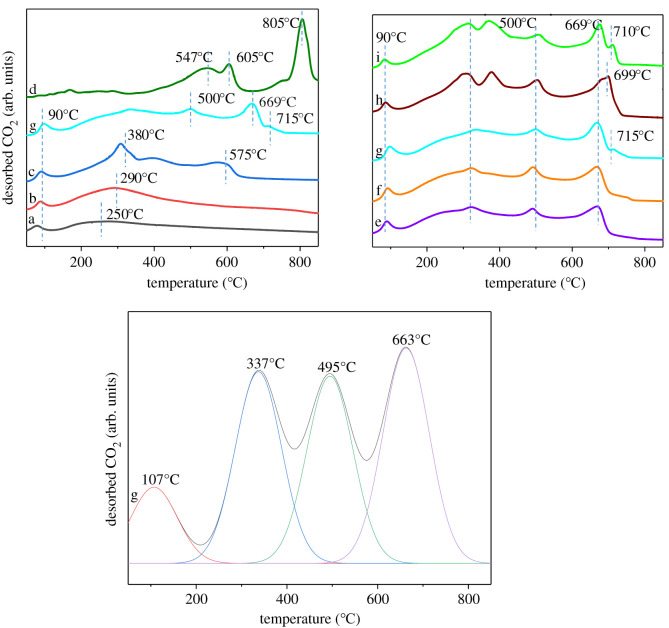
CO_2_-TPD profiles of (a) γ-Al_2_O_3_, (b) La/γ-Al_2_O_3_, (c) KF/γ-Al_2_O_3_, (d) KF/La_2_O_3_, (e) KF-15La/γ-Al_2_O_3_, (f) KF-20La/γ-Al_2_O_3_, (g) KF-25La/γ-Al_2_O_3_, (h) KF-30La/γ-Al_2_O_3_, (i) KF-35La/γ-Al_2_O_3_.

The strength of the basic sites of the catalysts was investigated using CO_2_-TPD to understand the adsorption and desorption characteristics of CO_2_ on the basic sites of the catalysts. The CO_2_ desorption profiles are shown in figure 9.

The strength of basic is one of the most important indicators of solid base. With an increase in the interaction between the catalyst surface and CO_2_, the CO_2_ desorption temperature increased. Table 1 shows the CO_2_ desorption content. A desorption peak was observed at approximately 90°C, which could be attributed to the desorption of CO_2_ adsorbed in the γ-Al_2_O_3_ channel. As shown in figure 9d, no peak was observed at 90°C, which could be attributed to the extremely small specific surface area of KF/La_2_O_3_, lack of pore structure and weak physical adsorption of CO_2_. A desorption peak was observed at 250°C, which was attributed to the weak basicity of Al_2_O_3_ (figure 9a), and an additional peak was observed at 290°C, which could be attributed to the large radius of La, which increased the basicity of La_2_O_3_ compared with that of Al_2_O_3_. After the metal oxide was loaded with KF (figure 9c,d,g), the CO_2_ desorption peaks shifted toward high temperature in comparison with that of the metal oxide catalyst (figure 9a,b). Because F^−^ ion has a higher electronegativity than O^2−^ ion, the negative charge of lattice oxygen was drawn toward F^−^ ion [67]. Thus, the KF-promoted metal oxide (Al_2_O_3_ or/and La_2_O_3_) contained more strong basic sites, compared with that of the corresponding metal oxide. Furthermore, CO_2_ desorption peaks were observed at 500°C, 669°C and 715°C, indicating that the KF-25La/ γ-Al_2_O_3_ catalyst contained strong basic sites (figure 9g). This may be attributed to the formation of La_2_O_3_ and LaOF, and the fact that the atomic radius of La was larger than that of Al, thus facilitating the loss of electrons, which resulted in the generation of F^−^ anions. Consequently, the KF-25La/γ-Al_2_O_3_ catalyst exhibited a stronger Lewis base site and CO_2_ interaction, thus increasing the desorption temperature of CO_2_. KF/La_2_O_3_ exhibited the highest CO_2_ desorption temperature, indicating that KF/La_2_O_3_ has a super base site; however, the specific surface area of KF/La_2_O_3_ was very small, thus limiting its catalytic effect.

A CO_2_ desorption peak was observed at 669°C (figure 9e–i). With a decrease in La loading content below 25% (figure 9e,f), the CO_2_ desorption temperature decreased. This could be attributed to the fact that the La load was small and did not completely cover the surface of γ-Al_2_O_3_, thus enabling the combination of Al with the excess KF to form a weak Lewis base site. However, with an increase in the La load beyond 25% (figure 9g–i), the desorption temperature of CO_2_ increased. Consequently, a CO_2_ desorption peak was observed in the pattern of KF-25La/γ-Al_2_O_3_ at 715°C. This could be attributed to the fact that the surface of γ-Al_2_O_3_ was fully covered with La_2_O_3_, and a large number of KF interacted with La_2_O_3_ to form strong Lewis base sites, which is consistent with the XRD and BET results. This indicates that the KF-25La/γ-Al_2_O_3_ catalyst exhibited the strongest basicity and the best macrolactonization catalytic effect.

The desorption isotherm of KF-25La/γ-Al_2_O_3_ (figure 9g) can be divided into four small peaks. A weak alkali peak was observed at 107°C, which could be attributed to the medium-strong base peak at 337°C and 495°C, and a strong alkali peak was observed at 663°C. The two medium-strong alkali peaks could be attributed to the desorption of the CO_2_ adsorbed on the outer surface of the catalyst at 337°C and the desorption peak at 495°C could be attributed to the adsorption of CO_2_ inside the catalyst. The results of the CO_2_-TPD characterization clearly show that we obtained a solid superbase with a maximum CO_2_ desorption temperature of 715°C. This will be very favourable for the catalytic activity of our macrocyclization reaction.

Combining all the characterization results, we obtained solid superbases with a relatively uniform distribution of active sites, pore sizes ranging from 16.83 to 22.58 nm and specific surface areas ranging from 37.32 to 62.94 m^2^ g^−1^.

### 3.2. Effects of the reaction conditions

**Figure 10.**
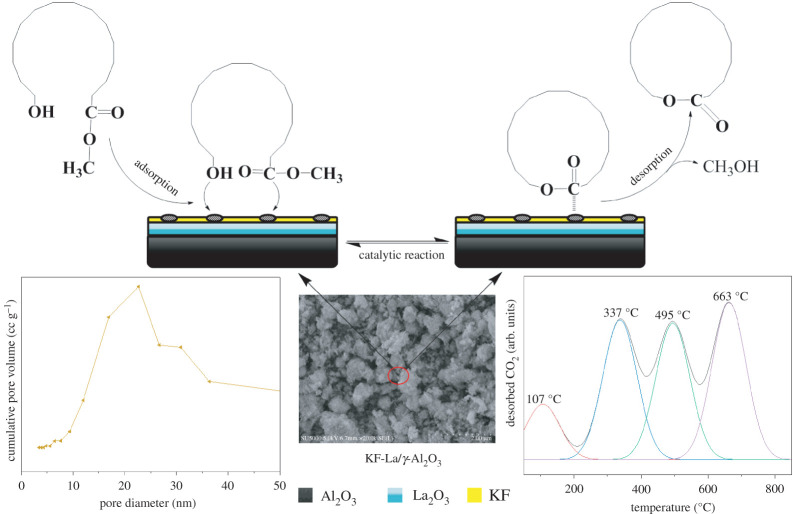
Proposed mechanism for macrolactonization of methyl 15-hydroxypentadecanoate with KF-La/γ-Al_2_O_3_ as catalyst.

Figure 10 shows the proposed mechanism of KF-La/γ-Al_2_O_3_-catalysed macrolactonization of methyl 15-hydroxypentadecanoate. Solid bases can accept protons or donate electrons when catalysing macrolactonization reactions. Assuming that the reaction took place on the surface of the catalyst, the first thing that occurs was that the alcohol and ester at both ends of the methyl 15-hydroxypentadecanoate were adsorbed on the active sites of the catalyst. Both the ester and alcohol then form intermediate on the active sites. The two ends of the intermediate react with each other to produce a cyclopentadecanolide molecule and a methanol molecule. This step was reversible, so it needed to be carried out under a vacuum, and the cyclopentadecanolide and methanol were continuously distilled out to ensure that the reaction was proceeding forward. After the reaction was completed, the active site of the catalyst catalysed other methyl 15-hydroxypentadecanoate reactions again.

#### 3.2.1. Effect of the catalyst type on the cyclopentadecanolide yield

**Figure 11.**
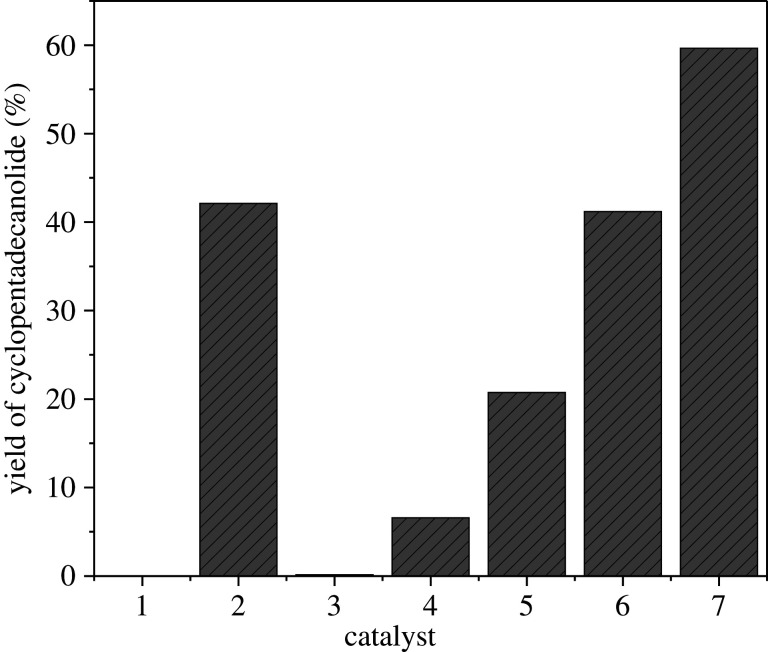
Comparison of the cyclopentadecanolide yields of the different catalysts. (1, no catalyst; 2, NaOH; 3, γ-Al_2_O_3_; 4, La/γ-Al_2_O_3_; 5, KF/γ-Al_2_O_3_; 6, KF/La_2_O_3_; 7, KF-La/γ-Al_2_O_3_).

Cyclopentadecanolide was synthesized via a catalytic reaction of methyl 15-hydroxypentadecanoate using a series of catalysts. These catalysts include NaOH, γ-Al_2_O_3_, La/γ-Al_2_O_3_, KF/γ-Al_2_O_3_, KF/La_2_O_3_ and KF-La/γ-Al_2_O_3_. The cyclopentadecanolide yield is shown in figure 11. The reaction was carried out using 0.5 g of the catalyst at a temperature and pressure of 190°C and 2 mbar, respectively, for 7 h. In the absence of a catalyst, the cyclopentadecanolide yield was 0%; however, the cyclopentadecanolide yield increased significantly after the addition of a catalyst. This indicates that the addition of catalysts significantly affects the macrolactonization of methyl 15-hydroxypentadecanoate.

When NaOH was used as a homogeneous catalyst to catalyse the macrolactonization of methyl 15-hydroxypentadecanoate, the cyclopentadecanolide yield was 41.29%. This could be attributed to the strong corrosivity of NaOH and the fact that it is a strong base that facilitated the saponification of methyl 15-hydroxypentadecanoate under high temperature and vacuum condition. However, the reaction system was prone to explosive boiling and the temperature control was difficult. Consequently, a large amount of water was required to wash the substrate at the end of the reaction. Therefore, different solid base catalyst was used to catalyse the macrolactonization. The γ-Al_2_O_3_-supporter exhibited the lowest yield (0.14%), which could be attributed to the lack of alkali active centres in γ-Al_2_O_3_ owing to its large specific surface area. When γ-Al_2_O_3_ was loaded with La_2_O_3_, the cyclopentadecanolide yield increased to 6.45%, which could be attributed to the low basicity of La_2_O_3_, which provided base sites for γ-Al_2_O_3_. When γ-Al_2_O_3_ was loaded with KF, the basicity and base content of the catalyst improved; consequently, a cyclopentadecanolide yield of 20.34% was achieved, which was confirmed by CO_2_-TPD.

The KF-La/γ-Al_2_O_3_ catalyst was obtained by sequentially loading La_2_O_3_ and KF on the surface of γ-Al_2_O_3_. Lanthanum oxide significantly affected the physical properties and catalytic activity of the catalyst. The La_2_O_3_ loaded on the surface of γ-Al_2_O_3_ interacted with KF to produce strong basic sites, while maintaining the large specific surface area and pore size of the catalyst. The addition of La facilitated the formation of mesoporous solid strong base on the surface of γ-Al_2_O_3_. Using mesoporous γ-Al_2_O_3_ with a pore size of 16.8 nm as the catalyst support facilitated the entry of the long-chain hydroxy esters (i.e. methyl 15-hydroxypentadecanoate) into the inner surface of the catalyst to enable its contact with the active sites inside. These results were obtained using BET and CO_2_-TPD. When the KF-(*ω*)La/γ-Al_2_O_3_ catalyst was used to catalyse the macrolactonization, a cyclopentadecanolide yield of 61.47% was achieved. However, when KF/La_2_O_3_ was used as the catalyst, the cyclopentadecanolide yield decreased, which could be attributed to the fact La_2_O_3_ has a small specific surface area regardless of its strong basicity, which restricted the reaction to the outer surface of metal oxides.

The cyclopentadecanolide yield of KF/La_2_O_3_ was lower than that of KF-(*ω*)La/γ-Al_2_O_3_ catalyst. These results indicate that the best catalyst for the synthesis of cyclopentadecanolide from methyl 15-hydroxypentadecanoate is a solid base catalyst with a large specific surface area, large pore size, strong basicity and a large amount of base active centre. Hence, KF-(*ω*)La/γ-Al_2_O_3_ was used as the catalyst for further investigations.

#### 3.2.2. Effect of La loading on the cyclopentadecanolide yield

**Figure 12.**
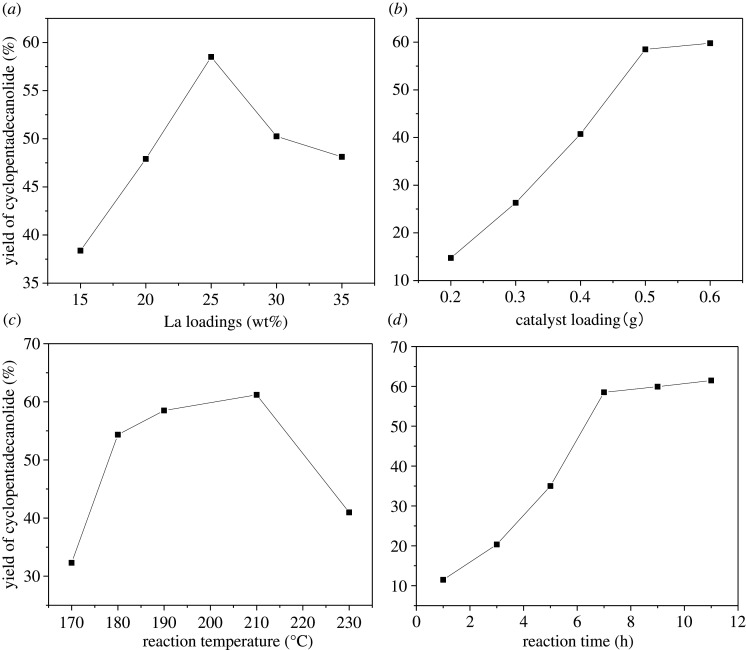
Effects of the reaction conditions: (*a*) La loading, (*b*) catalyst loading, (*c*) reaction temperature and (*d*) reaction time.

The effect of the La loading (15–35 wt%) of KF-ωLa/γ-Al_2_O_3_ on the macrolactonization of methyl 15-hydroxypentadecanoate was investigated, and the results are shown in figure 12*a*. The macrolactonization reaction was performed in a reduced pressure environment at 2 mbar. For this experiment, 0.5 g of the KF-(*ω*)La/γ-Al_2_O_3_ catalysts with various La loading content was used at 190°C for 7 h. With an increase in the La content from 15 to 25% w/w, the cyclopentadecanolide yield gradually increased. The low cyclopentadecanolide yield at low La loading content could be attributed to the fact that the La_2_O_3_ from the calcined La species did not completely cover the surface of the γ-Al_2_O_3_, resulting in fewer base sites and lower catalyst activity. With a further increase in the La content, the formed La_2_O_3_ layer interacted with KF to form more Lewis strong base sites. In addition, the excess La species filled the small pores and prevented the cleavage of the long-chain methyl 15-hydroxypentadecanoate and cyclopentadecanolide due to the prolonged diffusion in the small pores. This indicates that the basic strength of the catalysts increased with an increase in the La loading from the TPD-CO_2_ analysis. This indicates that a high basicity level facilitated this reaction. However, with a further increase in the La loading to 35 wt%, the cyclopentadecanolide yield decreased. With an increase in the La loading content, the coverage of the surface of the carrier by the La_2_O_3_ generated after calcination increased. Consequently, excess La_2_O_3_ agglomerated on the surface and pores of γ-Al_2_O_3_, resulting in a decrease in specific surface area of the supporter. These results are consistent with the XRD and BET results. These results indicate that the optimum La content that exhibit a moderate specific surface area, strongest basicity and highest cyclopentadecanolide yield, was 25% w/w.

#### 3.2.3. Effect of catalyst loading on the cyclopentadecanolide yield

The effect of the KF-25La/γ-Al_2_O_3_ content on the macrolactonization of methyl 15-hydroxypentadecanoate was examined, and the results are shown in figure 12*b*. The catalyst content was varied between 0.2 and 0.6 g, while the other parameters, including pressure, catalyst loading and temperature were kept at 2 mbar, 0.5 g and 190°C, respectively. In the absence of a catalyst, no cyclopentadecanolide was formed; however, cyclopentadecanolide was produced after the addition of a catalyst. This indicates that the addition of a catalyst reduced the activation energy of the reaction and accelerated the macrolactonization rate. In addition, we found that the catalyst content affected the cyclopentadecanolide yield. With an increase in the catalyst loading from 0.2 to 0.6 g, the cyclopentadecanolide yield increased from 14.74 to 59.79%. An insufficient catalyst content resulted in incomplete macrolactonization. With an increase in the catalyst content, the cyclopentadecanolide yield increased. With an increase in the KF-25La/γ-Al_2_O_3_ content beyond 0.5 g, the yield increased gradually, indicating that the optimum catalyst content is 0.5 g.

#### 3.2.4. Effect of temperature on the cyclopentadecanolide yield

Temperature is an important factor that affects macrolactonization. Therefore, the effect of the reaction temperature on the cyclopentadecanolide yield using KF-25La/γ-Al_2_O_3_ catalyst was investigated by varying the temperature from 170°C to 230°C, while other factors were kept constant, and the results are shown in figure 12*c*. With an increase in the temperature from 170°C to 210°C, the cyclopentadecanolide yield increased from 32.31% to 61.2%. However, the produced cyclopentadecanolide could not be extracted from the reaction system in time because the evaporation of glycerol decreases at a lower reaction temperature, thus increasing the resistance of the reaction equilibrium towards the direction of the product. Consequently, the cyclopentadecanolide yield decreased.

It was well known that an increase in the reaction temperature improves the reaction rate, and increases the evaporation of glycerine in the reaction system, thus enabling the acceleration of the reflux rate, the timely separation of the product and the shift in the reaction equilibrium toward the product. With an increase in temperature, the molecular motion increased, thus increasing the probability of collision between the hydroxyl groups and methoxy groups at the head and tail of methyl 15-hydroxypentadecanoate. Therefore, with an increase in the reaction temperature, the cyclopentadecanolide yield increased. However, with a further increase in the temperature to 230°C, the cyclopentadecanolide yield decreased. This could be attributed to the fact that extremely high temperatures trigger the saponification and carbonization of raw materials. In addition, excessive temperature affects the physical appearance of cyclopentadecanolide: for example, cyclopentadecanolide turns yellow. These results indicate that the optimum energy-saving temperature for the synthesis of good-quality cyclopentadecanolide is 190°C.

#### 3.2.5. Effect of the reaction time on the cyclopentadecanolide yield

The effect of the reaction time on the cyclopentadecanolide yield was investigated, and the result is shown in figure 12*d*. The reaction time was varied between 1 and 11 h, while other reaction parameters were kept constant. The change in the cyclopentadecanolide yield with a change in the reaction time is shown in figure 12*d*. With an increase in the reaction time, the cyclopentadecanolide yield increased from 11.48 to 61.47%. With an increase in the reaction time from 1 to 7 h, the yield increased rapidly owing to the relatively high concentration of raw materials. In addition, the product was removed from the reaction system in time and the reaction equilibrium moved forward, thus increasing the yield rapidly. However, with a further increase in the reaction time beyond 7 h, the reaction rate decreased. As the reaction time increased, the concentration of raw materials decreased. Consequently, the cyclopentadecanolide that was not taken out in time was retained in the system, thus increasing the resistance of the reaction equilibrium to a positive movement. In addition, the reaction system was easily carbonized under long-term high temperature conditions, and a large number of by-products generated in the system covered the active sites of the catalyst and reduced the catalytic activity of the catalyst. These results indicate that the optimum reaction time to achieve an energy-saving macrolactonization of methyl 15-hydroxypentadecanoate to cyclopentadecanolide is 7 h. The yield and content of cyclopentadecanolide obtained under this condition were 58.50% and 98.80%, respectively.

### 3.3 Reusability study of KF-25La/γ-Al_2_O_3_

**Figure 13.**
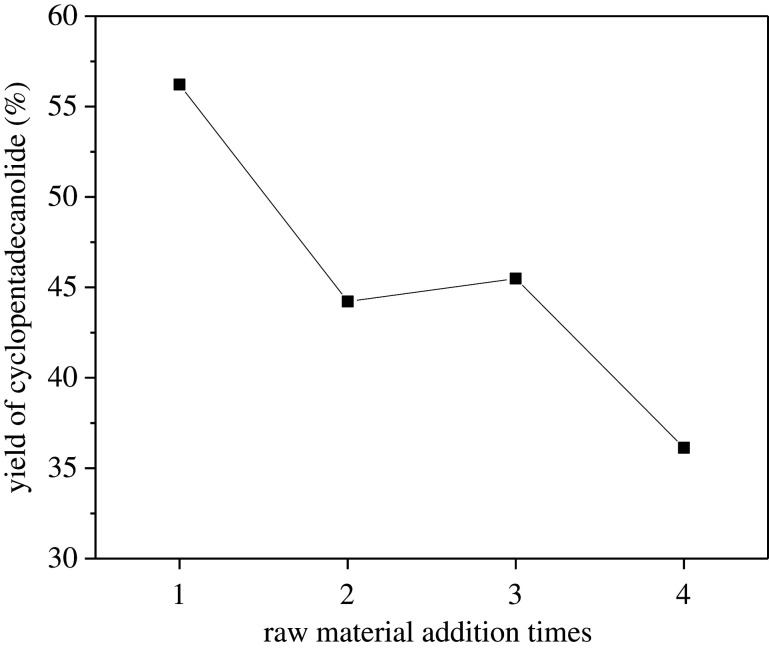
Continuous treatment of KF-La/γ-Al_2_O_3_.

Reusability is one of the main advantages of heterogeneous catalysts. Thus, KF-25La/γ-Al_2_O_3_ recycling was investigated with respect to macrolactonization reactions. After the reaction, the glycerol at the bottom of the reactor is distilled out, and the raw materials and fresh glycerol were added to react again. The cyclopentadecanolide yield obtained is shown in figure 13. The reaction yield decreased with the second and third addition of raw materials. After the fourth addition of raw materials, the total cyclopentadecanolide yield reduced to 36.13%, by which time the catalyst had turned brown in colour. This low yield may be due to the presence of a large amount of materials and by-products in the reaction system blocking the pores of the catalyst, as well as coking of the catalyst caused by high temperatures, which reduces the activity of the catalyst.

According to the study above, a solid strong base KF-La/γ-Al_2_O_3_ with a large pore size that was synthesized at a relatively low calcination temperature in this paper was facile and energy-conserved since the process of preparation. KF and La were sequentially loaded on γ-Al_2_O_3_ to achieve three main functions. Firstly, the lanthanum oxide covering the surface of γ-Al_2_O_3_ promoted the uniform dispersion of KF (SEM-EDX images). Secondly, KF reacted with La species to produce strong basic active sites (CO_2_-TPD). Lastly, KF and La species filled the small pores to prevent the prolonged diffusion of the large-molecular weight methyl 15-hydroxypentadecanoate through the small pores and cracking (BET). The catalytic performance of various types of catalysts was investigated, and the results revealed that the KF-25La/γ-Al_2_O_3_ catalyst exhibited the highest catalytic activity for the macrolactonization of methyl 15-hydroxypentadecanoate.

## 4. Conclusion

In this study, a solid base catalyst with large pore size was obtained under relatively mild calcination conditions using γ-Al_2_O_3_ as a support, which was used to catalyse the macrolactonization of methyl 15-hydroxypentadecanoate. The use of solid bases as catalysts relative to liquid catalysts reduces the risk of saponification of the raw materials. The reaction rectification method is adopted to shorten the reaction time (7 h). The whole reaction uses cheap and easily available glycerol as a solvent and an entrainer, and no other toxic and harmful reagents are used, which avoids subsequent treatment and does not pose a threat to the environment. The yield and content of cyclopentadecanolide obtained under 0.5 g KF-25La/γ-Al_2_O_3_, 190°C and 7 h were 58.50% and 98.80%, respectively. The macrolactonization was carried out at a higher concentration (0.33 mol l^−1^), and the obtained cyclopentadecanolide was of high purity, and no purification of the product was required. The catalyst can be re-used many times without treatment. KF-La/γ-Al_2_O_3_ solid base catalyst improves the overall synthesis efficiency and economy of cyclopentadecanolide, which can be extended to the synthesis of other macrolides.

## Data Availability

Data have been uploaded as electronic supplementary material [71].
